# Hypertriglyceridemia and Waist Circumference Predict Cardiovascular Risk among HIV Patients: A Cross-Sectional Study

**DOI:** 10.1371/journal.pone.0025032

**Published:** 2011-09-22

**Authors:** Peter M. Janiszewski, Robert Ross, Jean-Pierre Despres, Isabelle Lemieux, Gabriella Orlando, Federica Carli, Pietro Bagni, Marianna Menozzi, Stefano Zona, Giovanni Guaraldi

**Affiliations:** 1 School of Kinesiology and Health Studies, Queen's University, Kingston, Ontario, Canada; 2 Division of Endocrinology and Metabolism, School of Medicine, Queen's University, Kingston, Ontario, Canada; 3 Québec Heart Institute, Hôpital Laval Research Centre, Quebec City, Quebec, Canada; 4 Metabolic Clinic, Infectious and Tropical Disease Unit, Department of Medicine, University of Modena and Reggio Emilia, Modena, Italy; University of Cape Town, South Africa

## Abstract

**Background:**

Although half of HIV-infected patients develop lipodystrophy and metabolic complications, there exists no simple clinical screening tool to discern the high from the low-risk HIV-infected patient. Thus, we evaluated the associations between waist circumference (WC) combined with triglyceride (TG) levels and the severity of lipodystrophy and cardiovascular risk among HIV-infected men and women.

**Methods:**

1481 HIV-infected men and 841 HIV-infected women were recruited between 2005 and 2009 at the metabolic clinic of the University of Modena and Reggio Emilia in Italy. Within each gender, patients were categorized into 4 groups according to WC and TG levels. Total and regional fat and fat-free mass were assessed by duel-energy x-ray absorptiometry, and visceral adipose tissue (VAT) and abdominal subcutaneous AT (SAT) were quantified by computed tomography. Various cardiovascular risk factors were assessed in clinic after an overnight fast.

**Results:**

The high TG/high WC men had the most VAT (208.0±94.4 cm^2^), as well as the highest prevalence of metabolic syndrome (42.2%) and type-2 diabetes (16.2%), and the highest Framingham risk score (10.3±6.5) in comparison to other groups (p<0.05 for all). High TG/high WC women also had elevated VAT (150.0±97.9 cm^2^) and a higher prevalence of metabolic syndrome (53.3%), hypertension (30.5%) and type-2 diabetes (12.0%), and Framingham risk score(2.9±2.8) by comparison to low TG/low WC women (p<0.05 for all).

**Conclusions:**

A simple tool combining WC and TG levels can discriminate high- from low-risk HIV-infected patients.

## Introduction

Lipodystrophy is commonly observed among HIV-infected patients receiving antiretroviral therapy (ART) [1]. The HIV-lipodystropy phenotype is broadly characterized by generalized atrophy of subcutaneous adipose tissue (SAT) and/or hypertrophy of visceral adipose tissue (VAT) [2], [3] and increased ectopic fat storage [4], [5]. Not surprisingly, the majority of lipodystrophic HIV-infected patients exhibit the classic constellation of cardiovascular risk factors including visceral obesity, insulin resistance, dyslipidemia, hypertension, endothelial dysfunction [6], and subclinical atherosclerosis [7]. These patients are also at significantly greater prospective risk of developing type-2 diabetes [8] or experiencing myocardial infarction [9]. Nevertheless, only ∼50% of HIV-infected patients on ART ever develop lipodystrophy along with the associated cardiovascular and metabolic complications [1], [6]. Thus, there exists an unmet need for a simple yet accurate clinical tool to discern the high-risk from the low-risk HIV-infected patient.

In otherwise healthy populations, waist circumference (WC) has been advocated as a simple tool for identifying the high-risk, abdominally obese patient [10], [11]. However, the sensitivity of WC for identifying patients at cardiovascular risk may be limited due to its inability to distinguish VAT from abdominal SAT. Given that excess VAT has been shown to predict metabolic complications [12], [13], morbidity [14], and mortality [15] independent of other AT depots, including abdominal SAT, this distinction is not trivial. Lemieux and colleagues previously suggested that an elevated triglyceride (TG) level can be used as a marker of excess VAT storage and associated metabolic abnormalities in individuals with elevated WC [16]. This combination of an elevated WC and elevated TG levels, termed the *hypertriglyceridemic waist* phenotype, has since been proven a simple, but reliable screening tool for identifying non-HIV-infected individuals with increased visceral adiposity [17] and with elevated cardiovascular disease risk [18]. The objective of the current investigation was to evaluate the associations between a combination of WC and TG levels with body composition and cardiovascular risk among 2322 HIV-infected men and women.

## Methods

### Subjects

The current cross-sectional investigation includes 2322 HIV-infected patients (1481 men and 841 women) recruited at the metabolic clinic of the University of Modena and Reggio Emilia in Italy between 2005 and 2009. Patients from HIV clinics throughout Italy are referred or get direct access to the multidisciplinary treatment at the metabolic clinic where they obtain comprehensive metabolic and anthropometric diagnostic and therapeutic assessments for the presence of lipodystrophy and non-infectious comorbidities.

Inclusion criteria were serologically documented HIV-1 infection, age >18 years, at least 18 months of ART exposure, and, for patients with established diagnoses of dyslipidemia and hyperglycemia, stable lipid-lowering and diabetes therapy for at least 6 months. A signed informed-consent form to participate in this study was obtained from each patient. Patients were excluded if they reported or had documented evidence of any of the following cardiovascular conditions: previous myocardial infarction (n = 70), stroke (n = 11), percutaneous coronary angioplasty (n = 57), coronary artery bypass surgery (n = 8), or peripheral vascular disease (n = 19). The study was approved by local institutional review board (Comitato Etico Provinciale di Modena).

### Demographic Outcomes

Demographic and clinical data, including duration of HIV infection, prior diseases (Centers for Disease Control and Prevention classification), ART history, and lifestyle were obtained by medical chart review. Smoking was categorized into the following categories: non-smoker, light smoker (<10 cigarettes per day), or heavy smoker (≥10 cigarettes per day). Alcohol consumption was separated into heavy (≥20 g of ethanol per day), light (<20 g of ethanol per day), or none. Patients were considered to be physically active if they reported ≥4 hrs per week of any form of exercise.

### Cardiovascular risk factors

Insulin resistance (IR) was calculated using the homeostasis model assessment equation (HOMA-IR = [fasting insulin in mU/mL × fasting glucose in mmol/L)/22.5]. Total cholesterol, low-density lipoprotein (LDL) cholesterol, high-density lipoprotein (HDL) cholesterol, triglyceride, apolipoprotein A and B, glucose, and insulin levels were measured after an overnight fast. The presence of metabolic syndrome was diagnosed according to the clinical criteria proposed by the NCEP-Adult Treatment Panel III (NCEP-ATP-III) [19]. Finally, the Framingham risk score for each patient was calculated according to the equations proposed by the ATP-III [19].

### Anthropometrics

All patients underwent physical examinations at the same time point as blood was taken. Waist, hip, and thigh circumference, height and body weight were measured by a single operator. Waist circumference was measured in the narrowest point at the mid-way between the lowest rib and the iliac crest with the subject standing at the end of expiration. Hip circumference was taken at the largest point at the greater trochanters, while thigh circumference was measured mid-way between the hip and knee. The circumferences were calculated as the average of three measurements. Body mass index (BMI) was calculated as weight in kilograms divided by the square of height in meters.

### Body Composition

Whole body, trunk, leg and arm fat and lean mass were quantified using dual-energy x-ray absorptiometry (Lunar DPX-MD; Lunar Corporation, Madison, WI for scans before July 2007 and Hologic 4500 QDR Elite, Bedford, Massachussets for scans after that date). A single CT image at the level of the L4 vertebra was taken for quantification of VAT and abdominal SAT using a 64-multislice CT scanner (LightSpeed VTC; General Electric Medical System). Each CT image was analyzed using software application based on Advantage windows 4.4 GE medical system. Two radiologists assessed CT images for VAT and abdominal SAT. Agreement between the operators for VAT measurement was calculated on a subset of 40 scans demonstrated a high repeatability (r = 0.97, ß = 0.98). Similar results were observed for abdominal SAT.

### HIV History

Plasma HIV-1 RNA levels, CD4+ cell counts (most recent value and nadir), and cumulative exposure to non-nucleoside reverse-transcriptase inhibitors (NNRTI), nucleoside reverse-transcriptase inhibitors (NRTIs), and PIs were recorded. HIV load was categorized as undetectable if <40 copies/mL were present. Previous AIDS diagnosis was defined according to the Centers for Disease Control and Prevention category C. Lipodystrophy was defined using HIV Outpatient Study (HOPS) definition, with anthropomorphic categorizations of lipoatrophy, lipohypertrophy, and mixed form, as previously described [20].

### Patient categorization

Within each gender, patients were separated into 4 distinct groups based on their WC and TG levels: low WC/low TG, low WC/high TG, high WC/low TG, and high WC/high TG. For men, the cut-off values for WC and TG were ≥90 cm and ≥2.0 mmol/L, respectively, while those for women were ≥85 cm and ≥1.5 mmol/L, respectively. These cut-off values have previously been shown to provide the optimal sensitivity and specificity in identifying cardiovascular risk among men and women [16], [21].

### Statistical Analysis

Differences in all variables were calculated between the four WC and TG level groups. Differences in categorical variables were analyzed using Chi-square test. Differences in means of continuous variables with a normal distribution were analyzed using ANOVA, followed by Bonferroni post-hoc testing. Where continuous data was not normally distributed, a Kruskal-Wallis test was performed to assess differences between group medians, followed by post-hoc testing using the Mann-Whitney test. Age and physical activity were included as covariates in the analyses of anthropometric, body composition, and cardiovascular risk factors. A p value <0.05 was considered statistically significant. Statistical analyses were performed using STATA 10.1 Intercooled version for Mac, StataCorp, Collage Station, TX, USA.

## Results

The demographic, anthropometric, body composition and cardiovascular risk factors for the 1481 HIV-infected men and 841 women grouped according to their WC and TG levels are presented in Tables 1 and 2, respectively. Men in the high WC groups were approximately 3 years older than those in the low WC groups (p<0.05). Additionally, the men with a high TG/high WC were less active (29.0% physically active) than men in all other groups (range: 35.5–44.5%; p<0.05). A similar pattern was observed among the women, with the high TG/high WC group being older (44.1±7.8 versus 42.3±5.7 years, respectively; p<0.05) and less active than the low TG/low WC group (22.2 versus 33.5% physically active, respectively; p<0.05). No differences in smoking or alcohol consumption were observed in either gender. Accordingly, all comparisons of anthropometric, body composition, and cardiovascular risk outcomes statistically controlled for age and physical activity level.

**Table 1:** Demographics, anthropometrics, body composition, and cardiovascular risk factors among 1481 HIV-infected men.

	Low TG/Low WC	High TG/Low WC	Low TG/High WC	High TG/High WC
n (%)	308 (20.8)	611 (41.3)	166 (11.2)	396 (26.7)
**Demographics**				
Age, y, mean (SD)	44.2 (7.4)^a^	44.9 (6.5)^a^	47.2 (7.4)^b^	48.2 (7.5)^b^
Physical activity, n (%)	137 (44.5)^a^	217 (35.5)^b^	66 (39.8)^a, b^	115 (29.0)
Smoke (>10 cigs/day), n (%)	104 (33.8)^a^	211 (34.5)^a^	43 (25.9)^a^	119 (30.0)^a^
Alcohol consumption, n (%)	148 (48.1)^a^	277 (45.3)^a^	91 (54.8)^a^	215 (54.3)^a^
**Anthropometrics**				
BMI, kg/m^2^, mean (SD)	21.9 (2.2)^a^	22.2 (2.2)^a^	27.1 (3.6)^b^	27.4 (3.6)^b^
Waist circumference, cm, mean (SD)	81.3 (5.3)^a^	82.2 (4.9)^a^	98.1 (8.3)^b^	98.0 (8.1)^b^
Hip circumference, cm, mean (SD)	87.2 (4.2)^a^	86.7 (4.1)^a^	95.5 (5.6)^b^	95.0 (6.0)^b^
Thigh circumference cm, mean (SD)	46.6 (3.9)^a^	46.2 (3.7)^a^	50.5 (4.0)^b^	50.0 (4.1)^b^
**Body Composition**				
VAT, cm^3^, mean (SD)	94.9 (60.6)	117.7 (70.3)	178.8 (78.7)	208.0 (94.4)
SAT, cm^3^, mean (SD)	81.2 (58.7)^a^	75.5 (54.5)^a^	215.2 (114.8)	184.4 (98.7)
Total fat, kg, mean (SD)	7.6 (3.3)^a^	7.4 (3.7)^a^	16.4 (7.2)^b^	15.6 (6.5)^b^
Total lean, kg, mean (SD)	56.1 (35.2)^a, b^	53.5 (6.1)^a^	64.0 (49.0)^b^	61.9 (26.8)^b^
Arm fat, kg, mean (SD)	1.0 (0.6)^a^	1.1 (0.6)^a^	2.8 (1.8)^b^	2.6 (1.4)^b^
Arm lean, kg, mean (SD)	7.4 (1.4)^a^	7.3 (1.3)^a^	7.9 (1.4)^b^	7.9 (1.4)^b^
Leg fat, kg, mean (SD)	1.9 (1.3)	1.4 (0.9)	3.7 (2.5)	3.2 (2.1)
Leg lean, kg, mean (SD)	17.1 (2.4)^a^	17.1 (2.3)^a^	19.1 (3.0)^b^	19.3 (2.6)^b^
**Cardiovascular Risk Factors**				
Framingham risk, mean (SD)	5.8 (4.5)	9.5 (7.2)	7.6 (5.7)	10.3 (6.5)
Diabetes, n (%)	17 (5.5)^a^	43 (7.0)^a^	12 (7.2)^a^	64 (16.2)
Hypertension, n (%)	90 (29.2)	242 (39.6)^a^	80 (48.2)^a,b^	208 (52.5)^b^
Metabolic syndrome (ATP-III), n (%)	11 (3.6)	195 (31.9)	18 (10.8)	190 (48.0)
Fibrate use, n (%)	9(2.9)^a^	64 (10.5)^b^	4 (2.4)^a^	33 (8.3)^b^
Statin use, n (%)	8 (2.6)^a^	27 (4.4)^a^	5 (3.0)^a^	43 (10.9)
Triglycerides, mmol/L, mean (SD)	1.1 (0.3)^a^	3.2 (2.2)^b^	1.1 (0.2)^a^	3.1 (1.9)^b^
Total cholesterol, mmol/L, mean (SD)	4.3 (0.9)^a^	5.1 (1.2)^b^	4.4 (1.1)^a^	5.1 (1.3)^b^
HDL, mmol/L, mean (SD)	1.2 (0.4)^a^	1.0 (0.4)^b^	1.2 (0.4)^a^	1.0 (0.3)^b^
LDL, mmol/L, mean (SD)	2.7 (0.8)^a^	3.0 (1.0)**^b^**	2.7 (0.9)^a^	3.0 (1.0)^b^
ApoA1, mg/dL, mean (SD)	139.0 (29.0)^a^	133.8 (24.7)^b^	140.1 (25.6)^a,b^	135.2 (24.8)^a, b^
ApoB, mg/dL, mean (SD)	86.3 (22.8)^a^	109.2 (28.9)^b^	89.0 (24.8)^a^	109.3 (26.2)^b^
HOMA-IR, mean (SD)	3.6 (8.6)^a^	4.2 (3.9)^a^	4.2 (2.8)^a^	6.2 (6.1)
Albuminuria, n (%)	22 (7.1)^a^	64 (10.5)^a^	13 (7.8)^a^	50 (12.6)^a^

**Note:**

Continuous variables are presented as mean (± SD), while rates are presented as n (%). Unless otherwise denoted, all groups are significantly different from one another (P<0.05). Groups marked by the same superscript letter are not different from one another (P>0.05).

Apo, apolipoprotein; ATP-III, adult treatment panel III; BMI, body mass index; CAC, coronary artery calcium; HDL, high-density lipoprotein; HOMA-IR, homeostasis model assessment of insulin resistance; IQR, inter quartile range; LDL, low-density lipoprotein; SD,standard deviation; VAT, visceral adipose tissue.

**Table 2:** Demographics, anthropometrics, body composition, and cardiovascular risk factors among 841 HIV-infected women.

	Low TG/Low WC	High TG/Low WC	Low TG/High WC	High TG/High WC
n (%)	284 (33.8)	245 (29.1)	145 (17.2)	167 (19.9)
**Demographics**				
Age, y, mean (SD)	42.3 (5.7)^a^	43.2 (6.8)^a,b^	43.3 (7.5)^a,b^	44.1 (7.8)^b^
Physical activity, n (%)	95 (33.5)^a^	65 (26.5)^a,b^	37 (25.5)^a,b^	37 (22.2)^b^
Smoke (>10 cigs/day), n (%)	83 (29.2)^a^	74 (30.2)^a^	30 (20.7)^a^	46 (27.5)^a^
Alcohol consumption, n (%)	122 (43.0)^a^	86 (35.1)^a^	63 (43.5)^a^	64 (38.3)^a^
**Anthropometrics**				
BMI, kg/m^2^, mean (SD)	20.5 (2.1)^a^	20.5 (2.0)^a^	25.4 (4.1)^b^	26.3 (4.2)^b^
Waist circumference, cm, mean (SD)	76.4 (5.1)^a^	76.8 (5.4)^a^	92.6 (7.8)^b^	93.7 (8.1)^b^
Hip circumference, cm, mean (SD)	85.0 (4.9)^a^	84.9 (4.3)^a^	94.1 (6.9)^b^	94.6 (8.2)^b^
Thigh circumference, cm, mean (SD)	44.0 (3.9)	42.7 (4.0)	47.0 (4.9)^a^	47.2 (5.8)^a^
**Body Composition**				
VAT, cm^3^, mean (SD)	72.9 (39.1)	91.4 (68.0)	139.2 (146.7)^a^	150.0 (97.9)^a^
SAT, cm^3^, mean (SD)	143.7 (84.8)^a^	128.2 (63.4)^a^	266.4 (122.6)^b^	258.3 (111.5)^b^
Total fat, kg, mean (SD)	10.8 (4.0)^a^	9.9 (3.7)^a^	19.2 (7.5)^b^	18.9 (7.6)^b^
Total lean, kg, mean (SD)	38.2 (3.9)^a^	38.8 (4.2)^a^	42.9 (5.1)^b^	43.9 (6.3)^b^
Arm fat, kg, mean (SD)	1.7 (0.8)^a^	1.6 (0.7)^a^	3.7 (2.1)^b^	3.6 (1.9)^b^
Arm lean, kg, mean (SD)	4.5 (0.7)^a^	4.7 (1.5)^a^	5.2 (1.0)^b^	5.4 (1.0)^b^
Leg fat, kg, mean (SD)	3.0 (1.8)	2.2 (1.4)	4.7 (2.7)^a^	4.2 (3.0)^a^
Leg lean, kg, mean (SD)	12.1 (1.6)^a^	12.2 (1.8)^a^	13.4 (1.9)^b^	13.7 (2.0)^b^
**Cardiovascular Risk Factors**				
Framingham risk, mean (SD)	1.5 (1.3)^a^	2.6 (2.6)^b^	1.4 (0.9)^a^	2.9 (2.8)^b^
Diabetes, n (%)	11 (3.9)^a^	9 (3.7)^a^	8 (5.5)^a,b^	20 (12.0)^b^
Hypertension, n (%)	41 (14.4)	60 (24.5)^a^	39 (26.9)^a^	51 (30.5)^a^
Metabolic syndrome (ATP-III), n (%)	6 (2.1)	62 (25.3)	20 (13.8)	93 (55.7)
Fibrate use, n (%)	6 (2.1)^a^	12 (4.9)^a,b^	3 (2.1)^a,b^	11 (6.6)^b^
Statin use, n (%)	4 (1.4)^a^	5 (2.0)^a^	5 (3.5)^a,b^	13 (7.8)^b^
Triglycerides, mmol/L, mean (SD)	1.0 (0.3)^a^	2.6 (1.3)^b^	1.0 (0.3)^a^	2.5 (1.2)^b^
Total cholesterol, mmol/L, mean (SD)	4.6 (1.2)^a^	5.1 (1.2)^b^	4.8 (1.1)^a,b^	5.4 (1.3)
HDL, mmol/L, mean (SD)	1.5 (0.5)^a^	1.2 (0.4)^b^	1.5 (0.4)^a^	1.2 (0.3)^b^
LDL, mmol/L, mean (SD)	2.7 (0.9)^a^	3.1 (1.0)^b,c^	2.9 (0.9)^a,b^	3.3 (1.1)^c^
ApoA1, mg/dL, mean (SD)	159.6 (33.0)^a,b^	147.7 (29.4)^c^	160.8 (30.4)^a^	150.3 (29.1)^b,c^
ApoB, mg/dL, mean (SD)	85.0 (24.2)^a^	107.7 (29.3)^b^	91.2 (23.7)^a^	112.8 (29.6)^b^
HOMA-IR, mean (SD)	3.0 (3.1)^a^	3.9 (3.4)^b^	4.0 (3.4)^a,b^	5.2 (5.9)^b^
Albuminuria, n (%)	16 (5.6)^a^	20 (8.2)^a^	11 (7.6)^a^	9 (5.4)^a^

**Note:**

Continuous variables are presented as mean (± SD), while rates are presented as n (%). Unless otherwise denoted, all groups are significantly different from one another (P<0.05). Groups marked by the same super-script letter are not different from one another (P>0.05).

Apo, apolipoprotein; ATP-III, adult treatment panel III; BMI, body mass index; CAC, coronary artery calcium; HDL, high-density lipoprotein; HOMA-IR, homeostasis model assessment of insulin resistance; IQR, inter quartile range; LDL, low-density lipoprotein; SD,standard deviation; VAT, visceral adipose tissue.

BMI, WC, hip and thigh circumference levels were comparable between men with the same WC category, but were significantly greater in the high WC than in the low WC groups (Table 1). The same was true among the women (Table 2), with the exception of a slightly smaller thigh circumference in the high TG/low WC than in the high TG/low WC group (42.7±4.0 cm and 44.0±cm, respectively; p<0.05).

**Figure 1:**
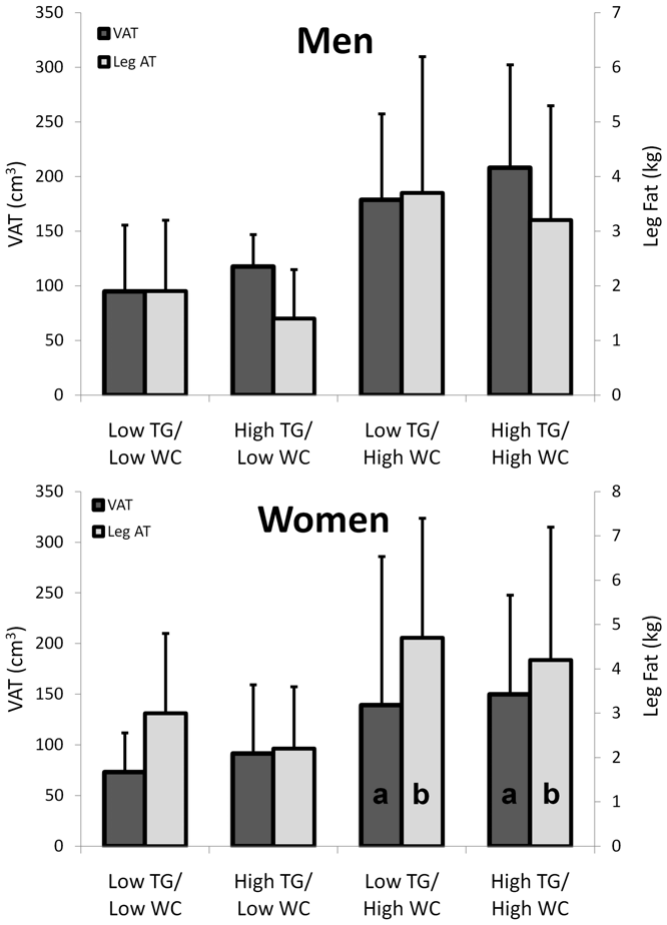
VAT and leg fat among HIV-infected men (top panel) and women (bottom panel) according to WC and TG category. Bars represent means, and the error bars represent the standard deviation. Bars with the same inset letter are not statistically different from one another (p>0.05). All other comparisons are significant (p<0.05).

In the men, VAT increased progressively from the lowest value among the low TG/low WC (94.9±60.6 cm^2^) to the highest value in the high TG/high WC group (208.0±94.4 cm^2^), meanwhile within each WC category, leg fat mass was 0.5 kg lower in the high TG versus the low TG groups (p<0.05, for all comparisons; Figure 1). In the women, VAT was highest among the high WC groups, but not different between the low TG/high WC and high TG/high WC groups (139.2±146.7 cm^2^ and 150.0±97.9 cm^2^, respectively; Figure 1). Additionally, in women with a low WC, leg fat was 0.8 kg lower in the high versus the low TG women (p<0.05). Aside from these differences, measures of total and regional body fat and lean mass were similar between groups within the same WC category but significantly higher in the high WC versus the low WC groups.

**Figure 2:**
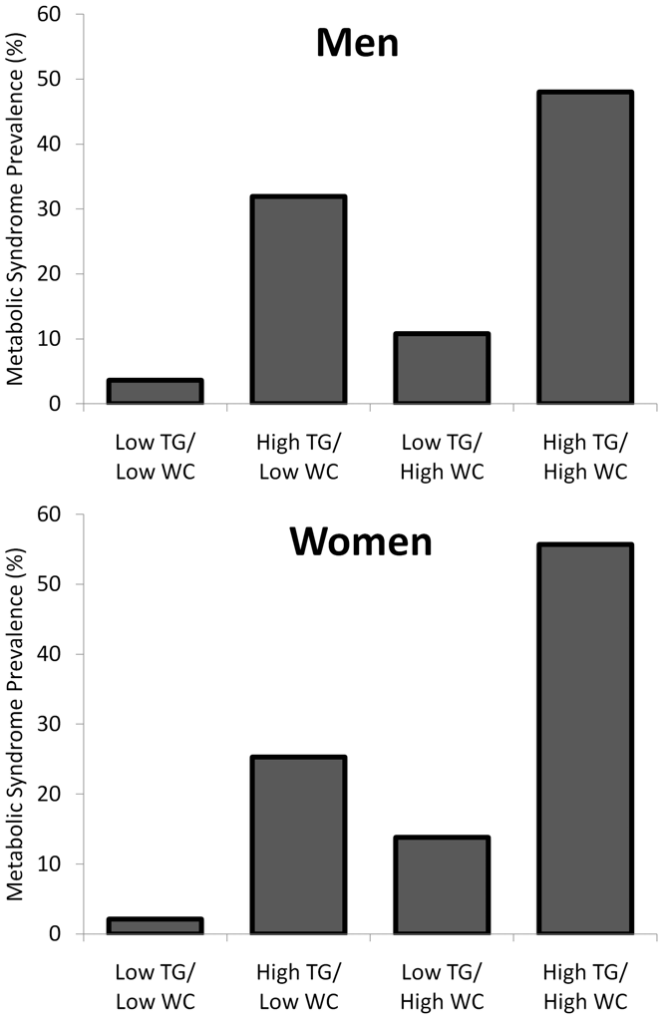
Metabolic syndrome prevalence among HIV-infected men (top panel) and women (bottom panel) according to WC and TG category. Bars represent prevalence (%).Bars with the same inset letter are not statistically different from one another (p>0.05). All other comparisons are significant (p<0.05).

**Figure 3:**
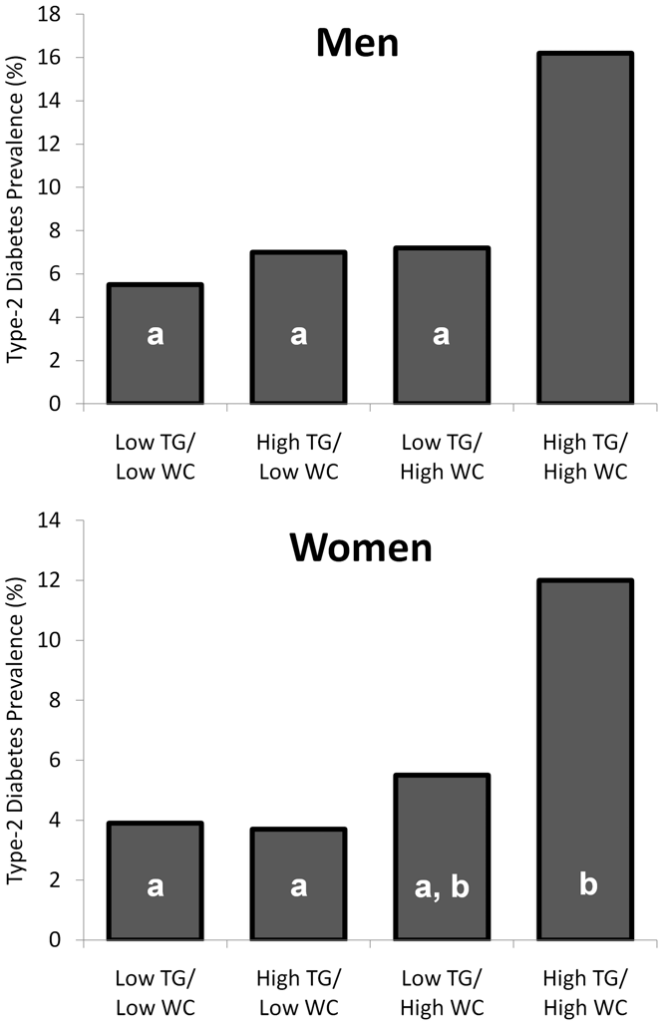
Type-2 diabetes prevalence among HIV-infected men (top panel) and women (bottom panel) according to WC and TG category. Bars represent prevalence (%).Bars with the same inset letter are not statistically different from one another (p>0.05). All other comparisons are significant (p<0.05).

In general, after control for age and physical activity level, cardiovascular risk was greatest among men in the high TG/high WC group (Table 1). For example, as illustrated in Figure 2, the prevalence of metabolic syndrome was significantly higher in the high TG/high WC men than in any other group (48.0% vs. range of 3.6–31.9%; p<0.05 for all comparisons). Additionally, the prevalence of type-2 diabetes was highest in the high TG/high WC group compared to all groups (16.2% versus 5.5–7.2%; p<0.05 for all comparisons) (Figure 3). Mean Framingham risk score was also significantly higher in the high TG/high WC men in comparison to all other groups (10.3±6.5 versus 5.8 to 9.5, respectively; p<0.05 for all comparisons; Table 1).

Globally, differences in the cardiovascular risk profile in the HIV-infected women according to WC and TG categories were less substantial than those observed in men (Table 2). Nevertheless, the prevalence of metabolic syndrome was significantly higher in the high TG/high WC women than in any other group (55.7% vs. range of 2.1 to 25.8%; p<0.05 for all comparisons). Additionally, after control for age and physical activity, high TG/high WC women were consistently at greater cardiovascular risk than low TG/low WC women. For example, diabetes rates in the high TG/high WC women (12.0%) were greater than the two low WC groups (3.7–3.9%; p<0.05). Mean Framingham risk scores were also significantly higher in the high TG/high WC than in the low TG/low WC women (2.9±2.8 vs. 1.5±1.3, respectively; p<0.05) as was the prevalence of hypertension (30.5 vs. 14.4%, respectively; p<0.05). Significant differences in cardiovascular risk between the high TG/high WC women and the two intermediate groups (high TG/low WC and low TG/high WC) were less consistent.

Outcomes pertaining to the history of HIV infection and treatment for the men and women are presented in Tables 3 and 4. Duration of exposure to any of the treatments did not differ between any of the groups within each gender. The nadir and the current CD4+ count were also comparable across the groups. Both men and women with a high TG/high WC were most likely to have a mixed form of lipodystrophy, marked by a combination of lipohypertrophy along with lipoatrophy (57.6% and 63.5%, respectively). Lastly, HIV-infected patients with low WC were more likely to experience lipoatrophy, while patients with a high WC were more likely to experience lipohypertrophy.

**Table 3:** HIV history among 1481 HIV-infected men.

	Low TG/Low WC	High TG/Low WC	Low TG/High WC	High TG/High WC
n (%)	308 (20.8)	611 (41.3)	166 (11.2)	396 (26.7)
Risk group				
IDU n (%)	109 (35.4)^a,b^	206 (33.7)^a,b^	62 (37.4)^a^	114 (28.8)^b^
MSM/Bisexual n (%)	135 (43.8)^a^	251 (41.1)^a^	49 (29.5)^b^	138 (34.9)^b^
Heterosexual n (%)	64 (20.8)^a^	154 (25.2)^a^	55 (33.1)^b^	144 (36.4)^b^
CD4+ Nadir median (IQR)	180.5 (67; 280)^a^	158 (59; 270)^a^	193.5 (97; 295.5)^a^	170.5 (64; 266)^a^
CD4+ Current median (IQR)	500 (371; 682)^a^	519.5 (364; 700)^a^	498 (379; 660)^a^	540 (374; 737)^a^
HIV Log_10_ VL mean (SD)	1.7 (1.7; 2.4)^a^	1.69 (1.7; 2.1)^a^	1.69 (1.7; 1.7)^a^	1.69 (1.7; 2.3)^a^
VL undetectable n (%)	177 (57.5)^a^	349 (57.1)^a^	106 (63.9)^a^	231 (58.3)^a^
Months of PI exposure median (IQR)	23 (0; 61)^a^	36 (10; 68)^a^	32 (1; 61)^a^	36 (8; 69)^a^
Months of NNRTI exposure median (IQR)	13.5 (0; 44)^a^	20 (0; 49)^a^	18 (0; 44)^a^	16 (0; 46.5)^a^
Months of NRTI exposure median (IQR)	94 (39; 131.5)^a^	106 (57; 136)^a^	88.5 (42; 122)^a^	102 (56.5; 135)^a^
Use of thymidine analogues n (%)	152 (49.4)^a^	280 (45.8)^a^	73 (44.0)^a^	167 (42.2)^a^
Use of ritonavir-boosed PIs n (%)	74 (24.0)^a^	165 (27.0)^a^	46 (27.7)^a^	122 (30.8)^a^
Lipodystrophy				
Lipoatrophy n (%)	169 (54.9)^a^	356 (58.3)^a^	19 (11.5)	27 (6.8)
Lipohypertrophy n (%)	6 (2.0)^a^	6 (1.0)^a^	38 (22.9)^b^	68 (17.2)^b^
Mixed form n (%)	42 (13.6)	133 (21.8)	63 (38.0%)	228 (57.6%)

**Note:** Unless otherwise denoted, all groups are significantly different from one another (P<0.05). Groups marked by the same superscript letter are not different from one another (P>0.05).

IDU, injection drug user; IQR, inter quartile range; HIV, human immunodeficiency virus; MSM, men who have sex with men; NNRTI, non-nucleoside reverse-transcriptase inhibitor; NRTI, nucleoside reverse-transcriptase inhibitor; PI, protease inhibitor; SD,standard deviation; VL, HIV RNA viral load.

**Table 4:** HIV history among 841 HIV-infected women.

	Low TG/Low WC	High TG/Low WC	Low TG/High WC	High TG/High WC
n (%)	284 (33.8)	245 (29.1)	145 (17.2)	167 (19.9)
Risk group				
IDU n (%)	92 (32.4)^a^	80 (32.7)^a^	37 (25.5)^a,b^	37 (22.2)^b^
Heterosexual n (%)	192 (67.6)	165 (67.4)	108 (74.5)	130 (77.8)
CD4+ Nadir median (IQR)	182 (80; 252)^a^	149.5 (52; 242)^a^	174 (77; 282)^a^	180.5 (61; 243)^a^
CD4+ Current median (IQR)	494.5 (369; 654)^a^	528 (366; 712)^a^	477.5 (359.5; 637.5)^a^	545 (375; 742)^a^
HIV Log_10_ VL mean (SD)	1.7 (1.7; 2.0)^a^	1.7 (1.7; 2.3)^a^	1.7 (1.7; 2.6)^a^	1.7 (1.7; 2.0)^a^
VL undetectable n (%)	186 (65.5%)^a^	143 (58.4%)^a^	84 (57.9%)^a^	98 (58.7%)^a^
Months of PI exposure median (IQR)	26.5 (0; 60)^a^	34 (4; 74)^a^	28 (0; 53)^a^	44 (15; 76)^a^
Months of NNRTI exposure median (IQR)	20 (0; 47)^a^	15 (0; 41)^a^	14 (0; 58)^a^	16 (0; 42)^a^
Months of NRTI exposure median (IQR)	106 (60.5; 137)^a^	112 (78; 148)^a^	102 (47; 138)^a^	106 (64; 144)^a^
Use of thymidine analogues n (%)	134 (47.2)^a,b^	132 (53.9)^a^	62 (42.8)^b^	82 (49.1)^a,b^
Use of ritonavir-boosed PIs n (%)	61 (21.5)^a^	64 (26.1)^a^	27 (18.6)^a^	41 (24.6)^a^
Lipodystrophy				
Lipoatrophy n (%)	114 (40.1)^a^	108 (44.1)^a^	9 (6.2)^b^	3 (1.8)^b^
Lipohypertrophy n (%)	11 (3.9)^a^	5 (2.0)^a^	35 (24.1)^b^	33 (19.8)^b^
Mixed form n (%)	75 (26.4)	94 (38.4)	70 (48.3)	106 (63.5)

**Note:**

Unless otherwise denoted, all groups are significantly different from one another (P<0.05). Groups marked by the same superscript letter are not different from one another (P>0.05).

IDU, injection drug user; IQR, inter quartile range; HIV, human immunodeficiency virus; NNRTI, non-nucleoside reverse-transcriptase inhibitor; NRTI, nucleoside reverse-transcriptase inhibitor; PI, protease inhibitor; SD,standard deviation; VL, HIV RNA viral load.

## Discussion

In the current study of 2322 HIV-infected patients, the combination of WC and TG levels was shown to discriminate high-risk from low-risk HIV-infected men and women. For instance, HIV-infected men with both high WC and high TG levels exhibited the most deleterious form of lipodystrophy, characterized by high levels of VAT combined with relatively low levels of thigh fat. Additionally, these men presented with greater deteriorations in cardiovascular health, experiencing the highest prevalence of metabolic syndrome, diabetes, hypertension, along with insulin resistance, dyslipidemia, and elevated Framingham scores. Furthermore, women with a high TG and high WC had more VAT and a deteriorated cardiovascular profile in contrast to low TG/low WC women. On the other hand, HIV-infected men and women with a low WC and low TG levels appeared to be largely protected from both these body composition and cardiovascular consequences. Thus, the present findings suggest a utility of WC combined with TG levels as a simple clinical tool for discerning the severity of lipodystrophy and associated cardiovascular risk among HIV-infected men and women.

During the late 1990's attention was first drawn to the potential impact of ART on body composition when reports documented the development of dorsocervical AT hypertrophy among HIV-infected patients; a finding reminiscent of the classic *buffalo hump* of Cushing's syndrome [22]. The tendency for generalized fat wasting [23], and/or excess VAT accumulation [2], [3] among HIV patients was also noted, and considered to be largely attributable to protease inhibitor treatment. It is now established that HIV-infected patients, particularly those treated with any ART, commonly develop lipodystropy, characterized by generalized atrophy of SAT, and/or hypertrophy of VAT [6], along with increased ectopic fat storage in non-AT tissues [4], [5]. In this regard, it is interesting that in our study the combination of two routinely obtained clinical measures, namely WC and TG, clearly identified HIV-patients with the most deleterious combination of body composition changes. Specifically, the men in the current study who met the criteria of the hypertriglyceridemic waist phenotype had the most VAT. Conversely, the same patients also had less abdominal SAT and thigh fat in comparison to their BMI- and WC-matched but low-TG counterparts. Thus, as depicted in Tables 3 and 4, both HIV-infected men and women with a high TG/high WC were most likely to have a mixed form of lipodystrophy (57.6% and 63.5%, respectively), marked by a combination of lipohypertrophy and lipoatrophy.

Along with the detrimental body composition changes, many lipodystrophic HIV-infected patients develop insulin resistance, dyslipidemia, hypertension, endothelial dysfunction [6], type-2 diabetes [8] and cardiovascular disease [9]. Nevertheless, there exists marked heterogeneity in cardiovascular risk among HIV-infected patients, as only 1 in 2 HIV-infected patients on ART ever develop these body composition and cardiovascular and metabolic complications [1], [6]. Thus, the identification and treatment prioritization of HIV-infected individuals at highest cardiovascular risk remains an ongoing challenge in the field. The results of the current study indicate that the combined use of WC and TG levels may be useful in this regard. Indeed, HIV-infected men and women with the hypertriglyceridemic waist phenotype were distinct from their low-risk HIV-infected counterparts, not only in terms of body composition, but also cardiovascular risk. Specifically, men with a high WC and high TG levels exhibited the highest rates of diabetes, hypertension, metabolic syndrome, along with the highest Framingham risk scores, as well as the most pronounced insulin resistance. Women with a high TG and high WC had the highest prevalence of metabolic syndrome, the highest total cholesterol levels, and were consistently at greater cardiovascular risk in comparison to the low TG/low WC groups.

The concept of the hypertriglyceridemic waist phenotype was first suggested over a decade ago, when it was observed that an elevated TG level can be used as a marker of excess VAT storage and associated metabolic abnormalities in otherwise healthy patients with elevated WC [16]. Subsequently, this phenotype has been proven a simple, but reliable screening tool for identifying non-HIV patients with increased visceral adiposity [17] and elevated cardiovascular disease risk [18]. The findings of the current study extend the utility of the hypertriglyceridemic waist phenotype beyond the general population to the identification of cardiovascular risk among ART-treated HIV-infected patients, particularly men.

Due to the cross-sectional design of the current investigation we were unable to test the utility of the hypertriglyceridemic waist phenotype in the prediction of prospective disease risk among HIV-infected patients; a potential focus of future research. Since patients in this study were specifically referred to the metabolic clinic from HIV clinics throughout Italy, it is possible that their disease severity was greater in comparison to the overall population of HIV patients. Thus, our findings may not be generalizable to all HIV patients. Another limitation of this study was the application of WC and TG thresholds which were established in an otherwise healthy population [16]. On the other hand, a major strength of the current study is the large sample size, which permitted the separate analysis of HIV-infected men and women. Further, homogeneity among the groups in demographic factors and HIV history provides added confidence in the results reported not being influenced by potential confounders.

In conclusion, the current study observed that the combination of WC and TG levels can clearly and consistently discriminate the high-risk from the low-risk HIV-infected patient. Indeed, HIV-infected men and women meeting criteria for the hypertriglyceridemic waist phenotype exhibited a more deleterious form of lipodystrophy and had elevated levels of cardiovascular risk factors in contrast to their low-risk counterparts. These findings suggest that a WC ≥90 cm and TG ≥2.0 mmol/L among HIV-infected men and a WC ≥85 cm and TG ≥1.5 mmol/L among HIV-infected women discriminates the high-risk from the low-risk patient in terms of body composition as well as cardiovascular risk.
